# A Heating-Assisted Direct Ink Writing Method for Preparation of PDMS Cellular Structure with High Manufacturing Fidelity

**DOI:** 10.3390/polym14071323

**Published:** 2022-03-24

**Authors:** Kang Xu, Dongya Li, Erwei Shang, Yu Liu

**Affiliations:** 1 School of Mechanical Engineering, Jiangnan University, Wuxi 214122, China; 6190805080@stu.jiangnan.edu.cn (K.X.); 8202011416@jiangnan.edu.cn (D.L.); 7180832007@stu.jiangnan.edu.cn (E.S.); 2 Jiangsu Key Lab of Advanced Food Manufacturing Equipment and Technology, Jiangnan University, Wuxi 214122, China

**Keywords:** additive manufacturing, silicone rubber, heating-assisted, manufacturing fidelity

## Abstract

In response to the fact that most of the current research on silicone 3D printing suffers from structure collapse and dimensional mismatch, this paper proposes a heating-assisted direct writing printing method for commercial silicone rubber materials for preparing silicone foam with enhanced fidelity. In the experimental processes, the effects of substrate temperature, printing pressure, and printing speed on the filament width were investigated using a controlled variable method. The results showed the following: (1) the diameter of silicone rubber filaments was positively correlated with the printing pressure and substrate temperature, but negatively correlated with the printing speed; (2) the filament collapse of the large filament spaced foams was significantly improved by the addition of the thermal field, which, in turn, improved the mechanical properties and manufacturing stability of the silicon foams. The heating-assisted direct writing process in this paper can facilitate the development of the field of microelectronics and the direct printing of biomaterials.

## 1. Introduction

Silicone (polysiloxane) has been used for a variety of medical applications since its initial commercialization in 1946, mainly owing to its biocompatibility and thermal stability. Silicones have a very flexible backbone structure for alternating silicon and oxygen atoms, which allows for the rotation of methyl groups (or other organic groups) toward the surface of the polymer. As a result, a low tension/energy surface is generally created that does not react with the surrounding environment. Medical-grade silicones are used in phantoms, prosthetics, pharmaceuticals, lubricating injection devices, etc. 

Direct ink writing has had a great impact as a versatile, multi-material, multi-scale 3D printing means in many fields, including stretchable electronic devices [1,2], soft body robotics [3,4], biomedical implants [5,6], and smart composites [7,8,9]. A wide variety of materials are used for direct ink printing, such as conductive pastes [10], super elastomers [4], and hydrogels [11,12], which often possess very similar rheological properties, such as viscoelasticity and shear-thinning [13,14].

Currently, research on external physical field-assisted direct-writing printing has focused on material modification and laser fields, such as Zhongyi Ji et al. [15], who added photo-initiators to polydimethylsiloxane (PDMS) precursors without photo-solidification to make them highly efficient for UV curing and prepared high-precision structures with a feature size of 100 μm; Lewis’s group [16] added a near-infrared laser to a direct-writing printing system to design and fabricate planar and spatially independent metallic structures at the microscale using nano-silver paste as the printing paste; Daniel et al. [17] investigated the laser heating process of thermosetting silicone rubber using a direct writing platform combined with an NIR laser and explored the parameters affecting the tensile stress of the samples; Mohammadreza Riahi et al. [18] borrowed the SLA system molding concept, combined a CO_2_ laser with PDMS, investigated the relationship between parameters, such as curing temperature and laser radiation and curing layer thickness, and prepared microchannels with a minimum feature size of 260 μm.

However, the most common PDMS precursors do not have unsaturated bonds or groups for curing or cross-linking, resulting in a lack of photo-responsiveness and the inability to support molding due to their low viscosity, i.e., they do not have the rheological properties required for direct writing. Although some custom PDMS devices have photo-responsiveness or suitable rheological properties, their mechanical properties are significantly different from those of commercial PDMS devices, and this difference can even reach an order of magnitude in terms of tensile strength. Therefore, the 3D printing of PDMS devices remains a challenge, especially with generic methods and commercial material-based 3D printing.

## 2. Materials and Methods

### 2.1. Materials

The PDMS used in this experiment was Dowsil SE1700 (Dow Chemical Company, Midland, MI, USA); 3-butyn-1-ol (Sigma-Aldrich Chemistry, St. Louis, MO, USA) was used as a retarder to grow the workable time of the ink [19]. To facilitate the detachment of the samples from the substrate, the substrate was soaked with an ethanol solution containing 1% trichloro (1H,1H,2H,2H-perfluorooctyl) silane (Sigma-Aldrich Chemistry, St. Louis, MO, USA) for 12 h.

During the preparation of the material, the PDMS base, crosslinker, and retarder were loaded into the glue cup in the mass ratio of 100:10:1 and stirred in a planetary mixer (QM3SP2, ZYE Technology, Shenzhen, China) at 1500 rpm for 2 min. Then, the mixture was transferred to a 30 cc or 10 cc syringe (Nordson EFD, Westlake, OH, USA) for printing and centrifuged in a centrifuge (TG1650-WS, BIORIDGE, Shanghai, China) at 5000 rpm for 5 min for de-airing.

**Figure 1:**
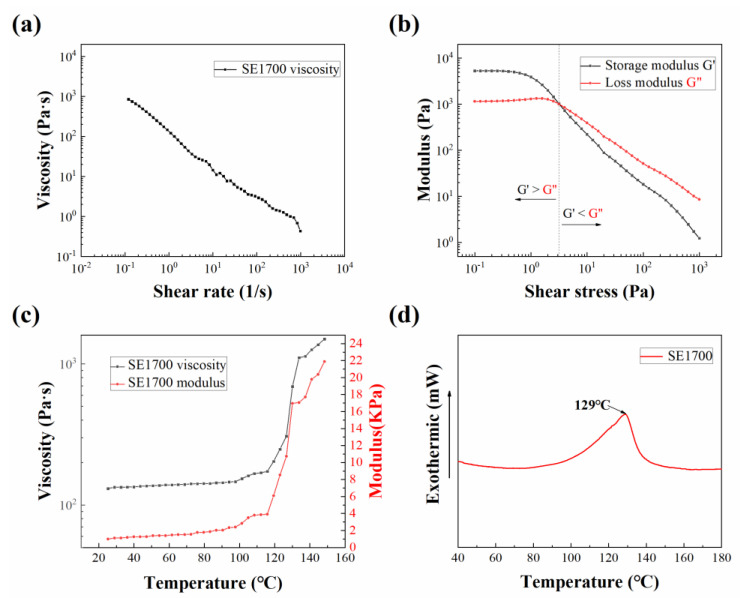
Characterization of properties of polydimethylsiloxane (PDMS): (**a**) viscosity-shear rate profile; (**b**) storage/loss-shear stress profiles; (**c**) temperature-viscosity/temperature-modulus profiles; (**d**) differential scanning calorimeter (DSC) profile.

After the material was prepared, the rheological properties were characterized. The relationship between the viscosity and the shear rate was measured in the shear rate range of 0.01–1000 s^−1^. It can be seen in Figure 1a that the viscosity of the PDMS decreased continuously as the shear rate increased, which means the paste possessed a shear-thinning property so that the high-viscosity PDMS could be extruded from the needle tip under shear conditions of pneumatic action. Stress scan tests were performed at an oscillation frequency of 1 Hz to obtain the storage modulus (G′) and loss modulus (G″) of the PDMS. As shown in Figure 1b, at low shear stress, G′ as greater than G″, when the PDMS exhibited solid-like behavior, which ensured that the printed PDMS structure did not collapse before curing; as the shear stress increased, above the gel-point (G′ = G″), G′ was less than G″, when the PDMS paste exhibited a liquid-like behavior.

Since the high-temperature platform was used in the printing process, the temperature-viscosity/temperature-modulus of the PDMS was characterized, and the results are shown in Figure 1c. As can be seen from the figure, during the process of temperature rise, the viscosity and the elastic modulus hardly changed in the stage of 25~100 °C; in the stage of 100~120 °C, the viscosity and elastic modulus showed a small increase; above 120 °C, the viscosity and elastic modulus increased substantially.

In addition, the crosslinking temperature of the PDMS was also characterized using a differential scanning calorimeter (DSC), and the characterization results are shown in Figure 1d. The test was performed with nitrogen as the protective gas and the temperature was increased from room temperature to 200 °C at a ramp rate of 10 °C/min. As shown in the figure, the cross-linking behavior of the PDMS appeared when the temperature was increased to 100 °C; until 129 °C, the peak cross-linking reaction rate was reached, which matched the curing behavior reflected by the previous temperature-viscosity profile (Figure 1c). Based on the temperature-viscosity profile, as well as the DSC profile, four temperature steps were specifically selected for subsequent sample printing: room temperature (≈25 °C), 90 °C, 120 °C, and 150 °C.

### 2.2. Simulation

The flow of paste in syringes was simulated by CFD (Computational Fluid Dynamics) using ANSYS Polyflow. In the simulation, the computational model was simplified to a 2D axisymmetric shape. The fluid was set up as a Herschel–Bulkley fluid, and the Picard iteration method is used to calculate the distribution of pressure, velocity, and shear rate within the flow field.

The heat conduction simulation in the foam was obtained by COMSOL Multiphysics software. In the simulation, an air domain with a side length of 50 cm was defined. A rectangle with a length and width of 25 cm and a thickness of 5 cm was added to the air domain as a copper heating substrate. Additionally, a silicone foam (silicone filament length of 8 mm and diameter of 0.5 mm) with 4 rods per layer and 10 layers in total was defined on the substrate. The upper surface of the substrate was set to 150 °C, and, in total, 100 time-intervals were set to obtain the temperature distribution inside the foam.

### 2.3. Experiments

**Figure 2:**
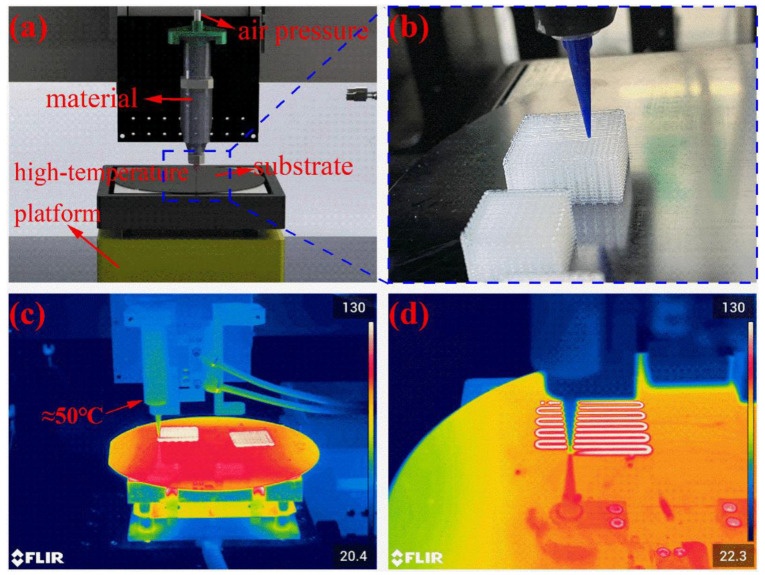
Heating-assisted direct writing 3D printing system: (**a**) overall view; (**b**) partial view; thermal imaging camera (E95, FLIR, Wilsonville, State of Oregon, USA) pictures of (**c**) overall view and (**d**) view of needle and sample.

The heating-assisted direct writing printing device consisted of a three-axis motion platform, a high-temperature platform module, a high-pressure gas extrusion module, and a needle tip, as shown in Figure 2a. The three-axis motion platform provided motion in the X/Y/Z directions for the needle tip to deposit the PDMS on the substrate placed on the high-temperature platform according to the set path. The high-temperature stage module used could provide a temperature output from 20 to 400 °C, with a temperature control accuracy of <1% in the heating area. Figure 2c,d and Figure S1 show the thermal imaging camera images during direct-write printing. The high-pressure gas extrusion module had an air pressure output range of 0.005 to 0.9 MPa and a needle tip with an inner diameter of 0.41 mm, which was connected to a syringe containing the PDMS and supplied with continuous air pressure at the rear end of the syringe. 

In this study, the temperature of the syringe had to be kept as close to room temperature as possible. However, due to the heat convection after heating the substrate, it was unavoidable that this temperature would rise somehow, as shown in Figure 2d, which was controlled at approximately 50 °C. According to Figure 1c, the rheological properties of the printed silicone ink did not change considerably and would help us in the extrusion of high-stability ink. 

### 2.4. Characterizations

The rheological properties of the printing paste were characterized using a rotational rheometer (ARES-G2, TA, New Castle, DE, USA); the cross-linking behavior of the PDMS was tested using a differential calorimetric scanner (Q2000, Waters, Milford, MA, USA); the internal structure of the silicone foams was characterized using computed tomography (x-eye SF160FCT, SEC, Suwon City, Korea); the morphology of the direct writing printed silicon foams was measured using an optical microscope (DVM6, Leica, Wetzlar, Germany); the compressive mechanical properties of the silicone foams with the same filament span printed on the different temperatures of the substrate was tested using a universal material testing machine (GJ211S, Qing Ji, Shanghai, China).

## 3. Results and Discussion

### 3.1. Determination of Printing Parameters

The main parameters involved in the regular direct writing process are printing pressure and printing speed. However, after adding a high-temperature platform, the material at the tip of the needle is affected by the thermal field of the substrate, resulting in a change in the viscosity of the material [20], which will affect the printing parameters. Therefore, this experiment mainly investigated the effects of printing pressure, printing speed, and substrate temperature on the filament width. 

**Figure 3:**
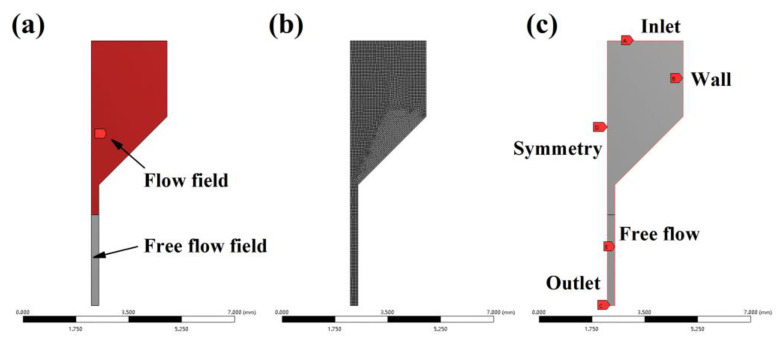
Fluid simulation model: (**a**) flow field and free flow field; (**b**) meshing; (**c**) boundary conditions.

**Figure 4:**
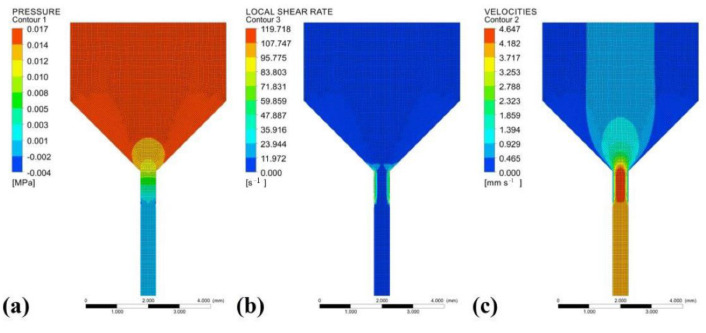
Results of fluid simulation: (**a**) pressure distribution; (**b**) shear rate distribution; (**c**) velocity distribution.

To investigate the flow of paste in the syringe, an axisymmetric 2D model, as shown in Figure 3a was established in ANSYS Polyflow, and the model was divided into a flow field and a free flow field. Figure 3b,c shows the mesh division results and boundary condition settings, respectively. According to the literature [21], the Herschel–Bulkley model was chosen for the viscosity and shear rate model in the simulation. The parameter settings are shown in Table 1. The results of the fluid extrusion simulation are shown in Figure 4. The pressure, shear rate, and velocity were kept constant at the syringe but varied more at the extrusion part. The larger pressure at the needle caused the paste to have a larger shear rate here, showing a shear-thinning characteristic, which facilitated the extrusion process of the paste.

An orthogonal experimental design was used in the parameter selection experiments to clarify the influence of printing speed (marked as A), printing pressure (marked as B), and substrate temperature (marked as C) on the printed filament width. Table 2 lists the factors and levels used in the orthogonal experiment. All the width data represent the average of five experimental results measured using optical microscopy. At the end of the experiments, the average values and ranges were calculated for each factor at each level, and the trend graphs were drawn, which reflect the influence of each factor level on the printed filament widths. 

Table 3 lists the orthogonal experiment parameters, results, and range analysis of the printed filament width experiment. The number in front of the brackets represents the level of the orthogonal experiment, and the number inside the brackets represents the experimental parameter corresponding to that level. The average I, II, III, and IV correspond to the average result of levels 1, 2, 3, and 4 for the different factors, respectively. The range is the difference between the maximum value and the minimum value of the experimental results of the four factors. Additionally, the greater the range, the greater the impact of the factor on the results. The orthogonal experimental design is a design method to study multiple factors and levels. It is based on orthogonality to select some representative points from the comprehensive test, which have the characteristics of “uniform dispersion, neat and comparable”. It can be concluded that the order that affects the printed filament width for the three factors is as follows: A—printing speed > B—printing pressure > C—substrate temperature.

We statistically measured the filament diameter by printing a silicone foam. The results are presented in Table 3. Under the same printing condition, the extruded filament diameter was kept stable with a very small variance of only approximately several microns. Based on these studies, our printing setup is believed to be feasible for sequential manufacturing. However, there are some limitations to this study. First, the printability and printing resolution of the pneumatic actuation method were limited by the viscosity of the silicone ink. In general, a higher viscosity will hamper the smoothness of the ink as it is extruded, meaning a finer filament cannot be obtained. Second, this proposed heating method from the substrate is insufficient for building a higher network due to the heating convection and conduction.

**Figure 5:**
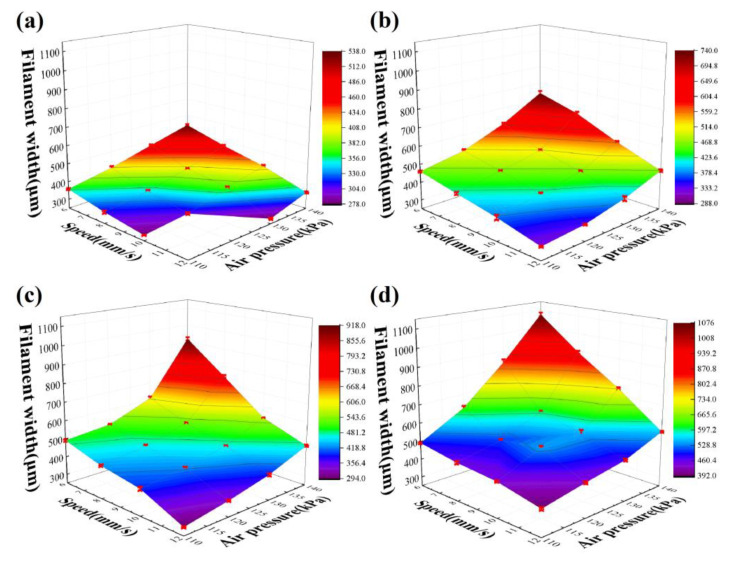
Heating-assisted direct writing parameters on: (**a**) room temperature substrate; (**b**) 90 °C substrate; (**c**) 120 °C substrate; (**d**) 150 °C substrate.

**Figure 6:**
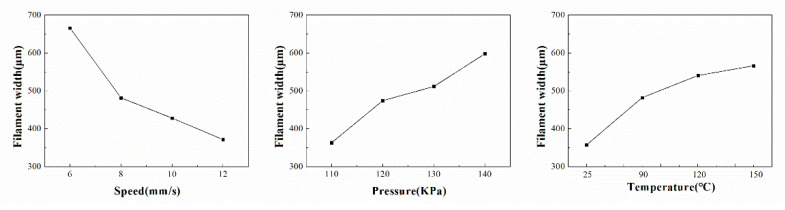
Trend graphs of the orthogonal experiment on the filament width experiment.

The experimental data are also shown in Figure 5. The X-axis is the printing speed; the Y-axis is the printing pressure; the *Z*-axis is the filament width; and the (a), (b), (c), and (d) plots represent the width characterization results under the substrate temperature of room temperature (≈25 °C), 90 °C, 120 °C, and 150 °C, respectively. The width increased with the increase in printing pressure, the decrease in printing speed, and the increase in substrate temperature. The same trends are also revealed in Figure 6.

In the printing parameter selection, if the material extrusion speed matched the substrate movement speed, it would obtain the suitable width of silicone rubber filaments (almost equal to the diameter of the needle tip). As observed by infrared thermography, the silicone rubber filaments need a certain amount of time to absorb the heat from the substrate to reach the state of a higher viscosity or modulus or even curing, so a lower printing speed was selected. The final printing parameters were determined, as listed in Table 4.

### 3.2. Experiment on the Collapse of Silicone Filaments

**Figure 7:**
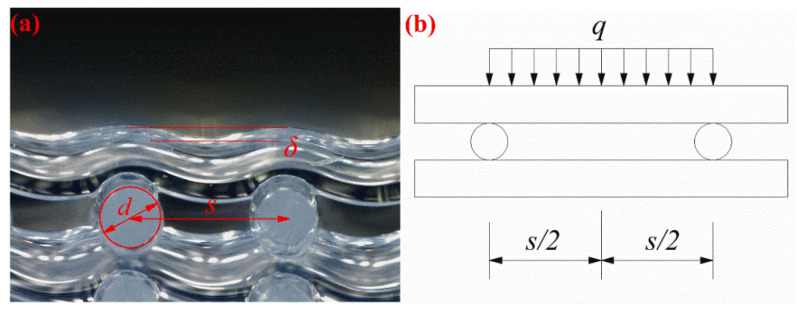
Structure of silicone foams: (**a**) schematic structural parameters of silicone rubber samples; (**b**) simplified mechanical model of filament stacking.

In the process of stacking silicone rubber filaments, the mechanical model can be seen as a simply supported beam model, as shown in Figure 7, and the self-weight of the silicone rubber filaments with span *s* can be regarded as a uniform load *q*. The collapse (*δ*) of the silicone rubber during the stacking process corresponds to the maximum deflection (ω_max_) of the simply supported beam, given by [22]:

(1) $$\omega_{\max}\;=\;-\frac{5qs^{4}}{384EI},$$

(2) $$I=\frac{\pi d^{4}}{64},$$

(3) $$\omega_{\max}=-\frac{5qs^{4}}{384EI}=-\frac{5qs^{4}}{384E\frac{\pi d^{4}}{64}}=k\frac{qs^{4}}{Ed^{4}},$$

where the negative sign indicates the downward direction; *q* is the homogeneous load (=0.25*ρg*_0_π*d*^2^); *ρ* is the paste density (≈1.13 g/cm^3^); *g*_0_ is the gravitational acceleration; *s* is the span of silicone rubber filaments; *E* is the elastic modulus of silicone rubber; *d* is the diameter of the silicone rubber filaments; *I* is the second area moment. The second area moment of the cylindrical rod is shown in (2). Substituting (2) into (1) can be simplified to obtain (3). The maximum deflection refers to the maximum displacement of the silicone rubber filament in the direction perpendicular to the axis (*δ* in Figure 7a), as viewed from the side of the sample.

In the silicone rubber direct writing process, the filament diameter *d* is a constant, the printed paste can be considered homogeneous, and the load *q* is a constant. *δ* is negatively correlated with the Young’s modulus of the material, which is positively correlated with the temperature. Therefore, it can be concluded that *δ* should be negatively correlated with the substrate temperature. Therefore, it can be inferred from (3) that, at a suitable amount of paste extrusion, the maximum collapse distance *δ* depends on the filament span *s* and material elastic modulus *E*. Therefore, the following can be speculated: (i) at a particular temperature, the material elastic modulus is almost constant, and the collapse distance *δ* can be considered to be positively correlated with the increasing span *s*; (ii) in a particular span of silicone foams, the material elastic modulus increases with an increase in substrate temperature (Figure 1c), so at this point, the collapse distance *δ* is negatively correlated with the increasing substrate temperature *T*.

**Figure 8:**
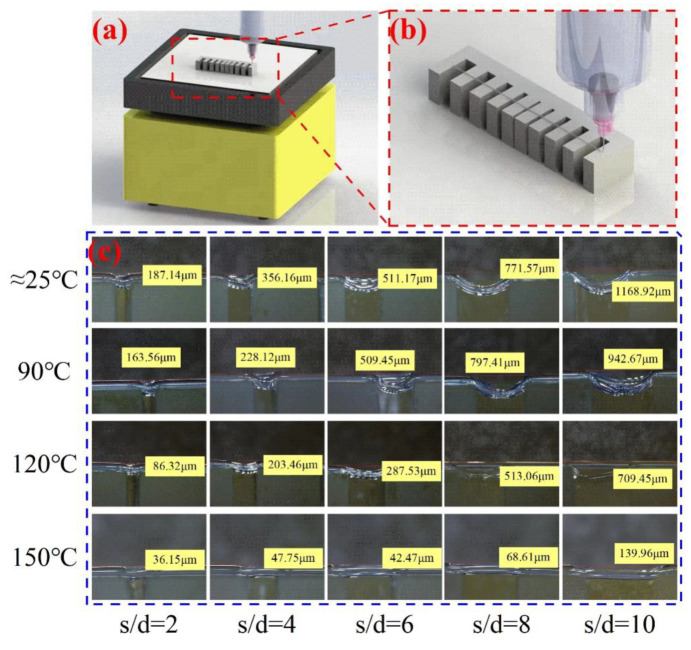
Experiment on the collapse of silicone rubber filaments: (**a**) schematic illustration of experimental equipment; (**b**) bracket with different gaps formed by digital light process (DLP); (**c**) characterization of silicone rubber filament collapse.

The mechanical properties of silicone foams depend on structural parameters, such as filament diameter *d*, span *s*, and needle lift height [23]. In this experiment, to investigate the collapse of each span under different temperature substrates, a bracket with different-width gaps was prepared using digital light process (DLP) technology (Figure 8), and the gap widths corresponded to the different spans in the silicone foams; during the experiment, different substrate temperatures were set and the bracket was used as the printing substrate to print silicone rubber filaments between the gaps. Finally, the collapse of the silicone rubber filaments was characterized under a microscope after printing.

**Figure 9:**
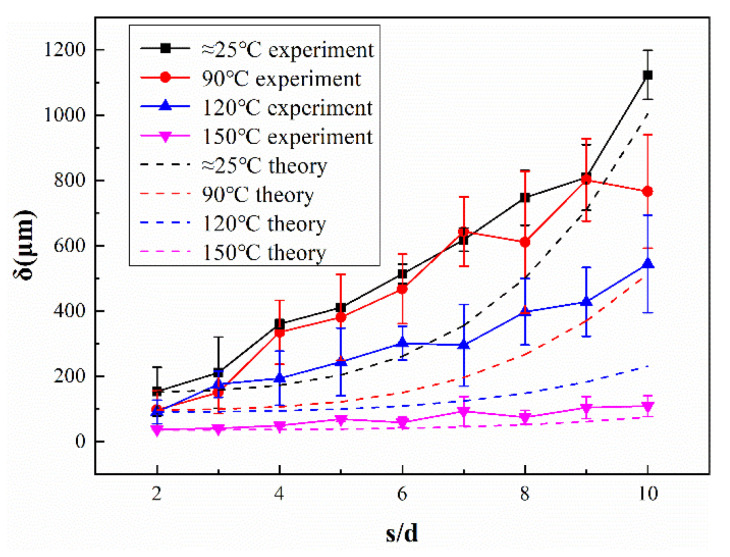
Collapse profile of silicon foams fabricated with different filament spans.

**Figure 10:**
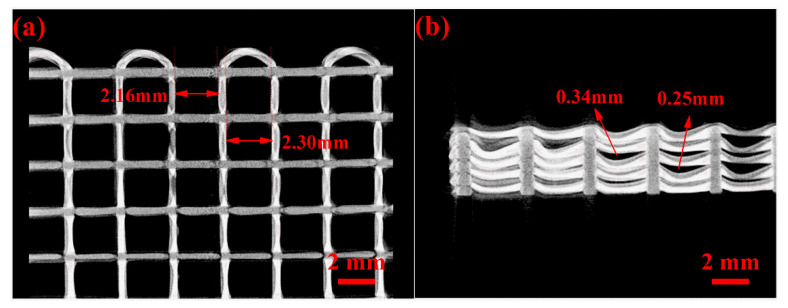
Computed tomography (CT) characterization images of silicone foams: (**a**) top view and (**b**) side view.

The collapse of the silicone rubber filament can be seen in Figure 8, Figure 9 and Figure 10: at the same substrate temperature, the collapse deteriorated as the filament span increased gradually, and the maximum collapse at room temperature could even exceed 1000 μm; at the same span, the collapse of the sample gradually decreased as the substrate temperature increased, and this situation gradually became more obvious as the span increased. In addition, when the span was small, the storage modulus of the uncross-linked material itself could “bear” the weight, which was barely influenced by the existence of the heating platform; while the span gradually increased, the modulus of the uncross-linked material was gradually unable to “bear” the increasing weight of the material. The collapse of the filaments assisted by the high-temperature platform was significantly improved due to the increase in the material viscosity and modulus.

In addition, a comparison between experimental and theoretical collapse is shown in Figure 9, where the dashed lines of different colors represent the theoretical collapse values of the silicone rubber filaments for different temperature substrates, which were calculated from (3) with the temperature-modulus profile, using the collapse value of the minimum span as the initial amount. As can be seen from the figure, the difference was enormous in all cases, except for the 150 °C substrates. Unlike the traditional way of calculating deflection, the silicone rubber filaments collapsed due to the weight itself after the stacking was completed due to a temporary lack of support during the stacking process, which is the main reason for the difference between the experiment and theory. It can be assumed that, as the span continues to increase, some other factors (e.g., collapse during stacking and the assistance of external thermal field) can be gradually ignored, and the experiments will be closer to the theory.

**Figure 11:**
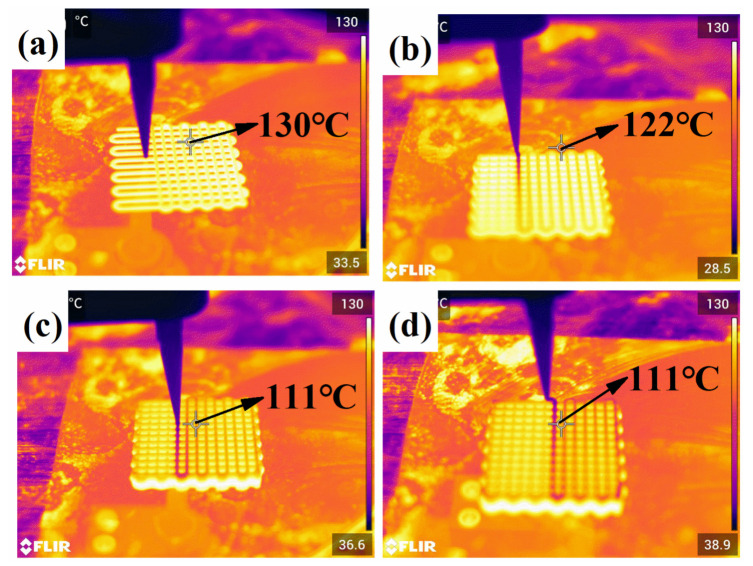
Thermal imaging camera picture during direct writing: (**a**–**d**) corresponds to the second layer, 4th layer, 8th layer, and 10th layer of the silicone foam, respectively. The figure shows that the temperature distribution of the first 10 layers is balanced. Additionally, the surface temperature of the highest layer (the 10th layer) is still above 110 °C.

**Figure 12:**
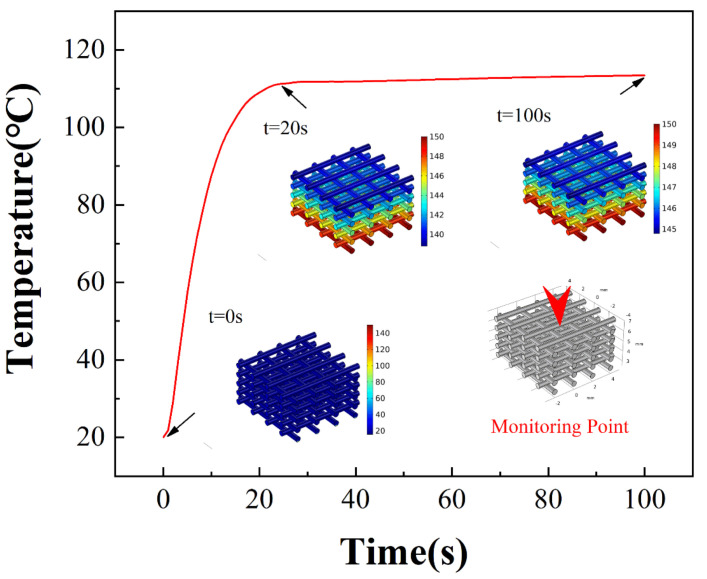
Temperature profile of silicone line in the top layer.

The limitation of this study is that silicone foams cured by the high-temperature substrate were based on heat conduction. Figure 11 shows the different surface temperatures at different layers, and the temperature was reduced by approximately 20 °C from layer 1 to layer 10. The heat conduction process simulated in COMSOL Multiphysics is shown in Figure 12. The temperature variation of the silicone line in the top layer was monitored. Additionally, the temperature of the top layer stabilized at approximately 110 °C after 20 s of heating. This is consistent with the thermal imaging camera characterization results. When a 30-layer foam was tested, as shown in Figure S1, the surface temperature was reduced to 70 °C, and our proposed heating method from the substrate lost its effectiveness in PDMS curing, while the thickness of samples increased. Therefore, this method is typically suitable for the preparation of foam samples with smaller heights.

### 3.3. Mechanical Compression Properties Testing of Silicone Foams

**Figure 13:**
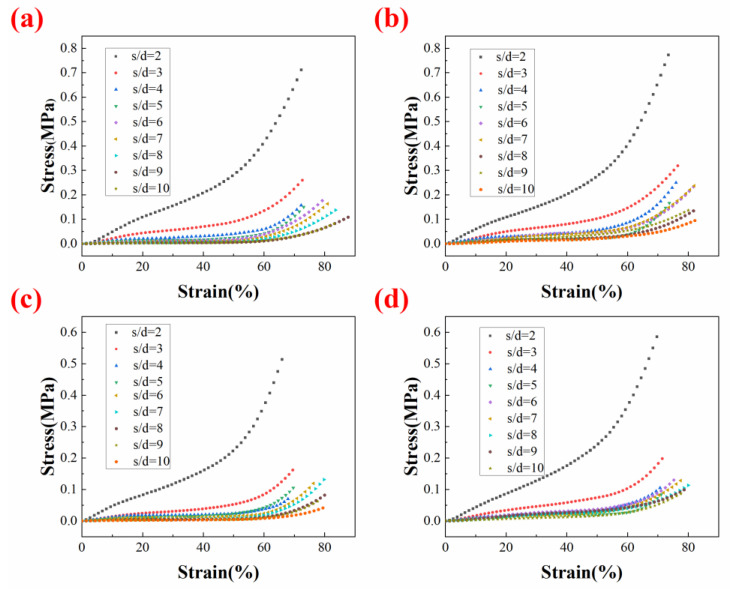
Compression stress–strain profiles of s/d = 2–10 samples at (**a**) room temperature substrate; (**b**) 90 °C substrate; (**c**) 120 °C substrate; (**d**) 150 °C substrate.

In this study, to show the performance of heating-assisted direct writing, the mechanical compression experiments were mainly based on the same span samples printed on different temperature substrates. Figure 13 shows the compression stress–strain profiles of the foams with different filament spans at room temperature substrate, 90 °C substrate, 120 °C substrate, and 150 °C substrate. Additionally, the foam strength decreased sharply when the span increased, so a silicon foam sample with s/d = 5 was selected for subsequent comparative experiments with both a certain span and mechanical strength. 

The addition of the external thermal field led to a change in the silicone foam curing process from the previous one-stage curing (oven curing) to the current two-stage curing (high-temperature platform-oven curing). To exclude the influence of the curing process on the mechanical properties, the same silicone foams were prepared on the 150 °C substrates and cured in the oven for different durations, and the stress–strain profiles are shown in Figure S2, among which the different curing processes had little influence on the mechanical properties of the samples and could be ignored in the subsequent comparison of the sample properties.

**Figure 14:**
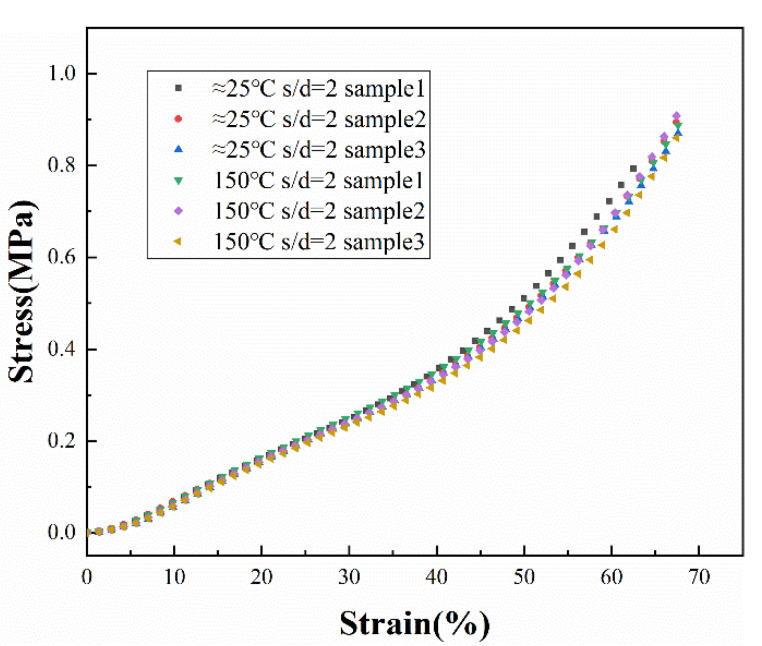
Stress–strain profiles of s/d = 2 samples.

**Figure 15:**
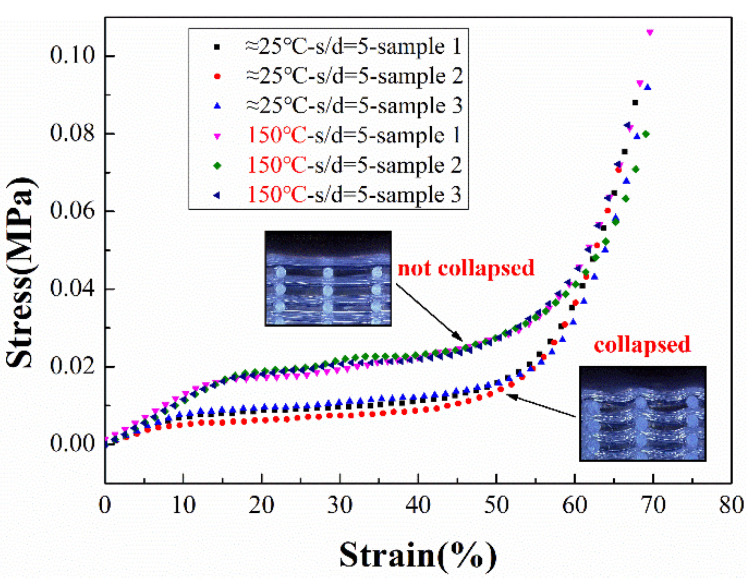
Stress–strain profiles of s/d = 5 samples.

In Figure 14 and Figure 15, the stress–strain profiles of the s/d = 2 and s/d = 5 samples printed on different temperature substrates can be seen from the graphs: (i) The s/d = 2 silicone foams had a higher number of stress columns within the same area compared to s/d = 5 foams, resulting in a higher strength in compression. (ii) In the case of s/d = 2, the structures of the silicone foams with and without the high-temperature platform were similar, and, therefore, exhibited similar mechanical properties. (iii) In the case of s/d = 5, the silicone foams manufactured with the assistance of a high-temperature platform exhibited higher strength and higher manufacturing stability due to the lower level of collapse within the sample, and the structure of the foam was less likely to buckle during compression.

## 4. Conclusions

In this paper, we developed a method to fabricate silicone foam devices with external thermal field-assisted direct writing, which was used to improve the filament collapse inside the foams and enhance the mechanical properties of the silicone foams. This study used SE1700 silicone rubber as the experimental material, obtained the printing parameters of heating-assisted silicone rubber direct writing by controlling the variables, and verified the advantages of heating platform-assisted direct writing. Compared with conventional direct writing, this study mainly explored the effects of substrate temperature, printing pressure, and printing speed on the diameter of silicone rubber filaments after adding the thermal field. Due to the change in viscosity and surface tension of the paste caused by the external thermal field, less printing pressure was required to obtain the same filament width at the same printing speed compared to conventional direct writing. The experimental results showed that: (i) the diameter of silicone rubber filaments was positively correlated with the printing pressure and substrate temperature; (ii) the diameter of silicone rubber filaments was negatively correlated with the printing speed. Finally, silicone foams with different spans were prepared using the above printing parameters, and their compression properties were tested. The experimental results show that the additional high-temperature platform effectively reduced the collapse between the layers of silicone foams and improved the strength and manufacturing stability of silicone foams at a larger filament span.

## Figures and Tables

**Table 1:** Parameters of Herschel–Bulkley model.

Parameters	Herschel–Bulkley
Yield stress (Pa)	764.01
Consistency coefficient (Pa·s)	86.997
Flow index	0.6259
Critical shear rate (s^−1^)	1.316

**Table 2:** Factor-level table selected in the orthogonal experiment.

Factor	A: Printing Speed (mm/s)	B: Printing Pressure (KPa)	C: Substrate Temperature (°C)
Level 1	6	110	≈25
Level 2	8	120	90
Level 3	10	130	120
Level 4	12	140	150

**Table 3:** Orthogonal experiment parameters, results, and range analysis.

No.	Factors			Results	
	A: Printing Speed (mm/s)	B: Printing Pressure (KPa)	C: Substrate Temperature (°C)	Filament Width (μm)	Error Bar (μm)
1	2 (8)	2 (120)	2 (90)	463.37	3.59
2	4 (12)	1 (110)	2 (90)	320.98	3.61
3	2 (8)	3 (130)	4 (150)	602.68	6.38
4	1 (6)	2 (120)	4 (150)	736.33	6.29
5	3 (10)	1 (110)	4 (150)	435.89	6.27
6	3 (10)	3 (130)	3 (120)	463.40	6.85
7	1 (6)	3 (130)	2 (90)	667.97	10.77
8	4 (12)	2 (120)	3 (120)	393.25	9.58
9	1 (6)	4 (140)	3 (120)	917.72	9.29
10	4 (12)	3 (130)	1 (≈25)	312.87	7.78
11	2 (8)	4 (140)	1 (≈25)	473.38	5.38
12	3 (10)	4 (140)	2 (90)	509.55	7.99
13	2 (8)	1 (110)	3 (120)	388.53	7.50
14	4 (12)	4 (140)	4 (150)	491.18	5.54
15	1 (6)	1 (110)	1 (≈25)	341.00	4.46
16	3 (10)	2 (120)	1 (≈25)	305.29	5.96
Average Ⅰ	665.755	363.77	358.135	-	-
Average Ⅱ	481.99	474.56	482.6375	-	-
Average Ⅲ	428.5325	511.73	540.725	-	-
Average Ⅳ	379.57	597.9575	566.52	-	-
Range	286.185	234.1875	208.385	-	

**Table 4:** Heating-assisted direct writing parameters.

Substrate Temperature	≈25 °C	90 °C	120 °C	150 °C
Printing pressure (KPa)	135	120	115	100
Printing speed (mm/s)	6			

## Data Availability

All the data presented in this work are available by contact with the corresponding author.

## Supplementary Materials

The following supporting information can be downloaded at: https://www.mdpi.com/article/10.3390/polym14071323/s1. Figure S1: Thermal imaging camera pictures during direct writing; Figure S2: Effect of curing process on mechanical properties of silicone foams.
