# Supplementary material for: A Heating-Assisted Direct Ink Writing Method for Preparation of PDMS Cellular Structure with High Manufacturing Fidelity
Source: Polymers (Basel). 2022 Mar 24;14(7):1323. doi: 10.3390/polym14071323 (PMC9002618; doi:10.3390/polym14071323)
Supplement: Supplementary file 1 [file polymers-14-01323-s001.zip › polymers-1577327-supplementary.pdf]

polymers

MDPI

Supplementary Information

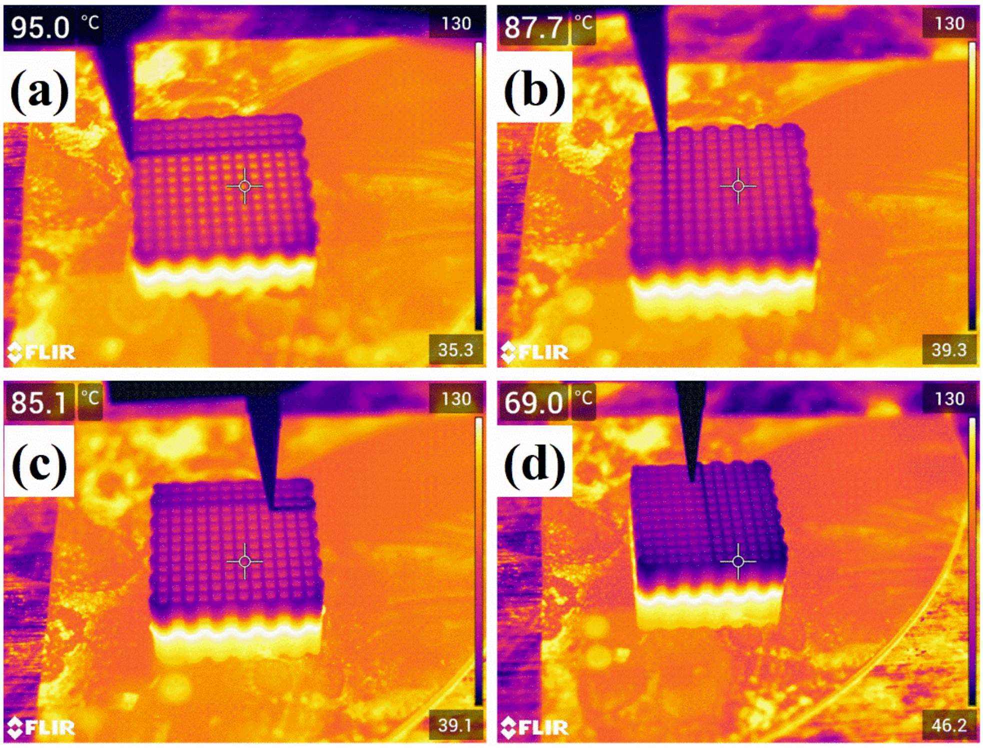

Figure S1. Thermal imaging camera pictures during direct writing: (**a**–**d**) show the thermal imaging camera pictures of the 15th, 20th, 25th, and 30th layer of the silicone foam, respectively.

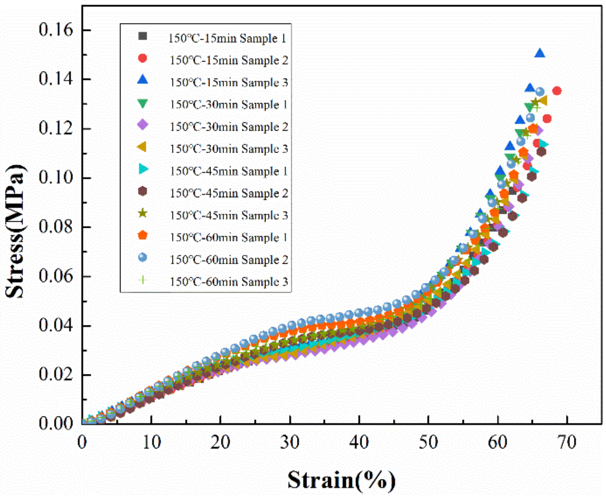

Figure S2. Effect of curing process on mechanical properties of silicone foams.
